# Perceptions of Parents on Management of Food Allergy in Children with Autism Spectrum Disorder (ASD) in Saudi Arabia

**DOI:** 10.3390/children10010048

**Published:** 2022-12-26

**Authors:** Talal E. Alhuzimi, Mudi H. Alharbi

**Affiliations:** 1 Department of Special Education, College of Education, King Saud University, Riyadh 11362, Saudi Arabia; 2 Clinical Nutrition Department, College of Applied Medical Sciences, Taibah University, Madinah 42353, Saudi Arabia

**Keywords:** allergens, autism children, parents, food, Saudi Arabia, prevalence, awareness, management of food allergy

## Abstract

Background: Food allergy is one of the most serious health concerns spread across the globe. Its awareness and management are undervalued, especially in children with autism spectrum disorders (ASD). The aim of this study is to explore the perception of parents of autistic children from Saudi Arabia on the prevalence, knowledge, awareness and management of food allergens. Methods: A cross-sectional exploratory self-administrated online survey was conducted in Saudi Arabia, where 125 parents of autistic children voluntarily took part in the survey from April to August 2022. Results: This study indicates that less than one-fourth of autistic children suffering from food allergies, while most of them are allergic to proteins mainly. Examination of the knowledge level of these parents regarding food allergies, depending upon the score of correct answers given by them, showed that a majority of parents had a moderate level of knowledge on food allergy. Even though there was awareness about food allergens, the use of medical interventions was not employed much. However, the parents were mindful of food labeling and found it to be useful in avoiding known food allergies. Mediating effects of food allergy were observed in the relationship between food allergy knowledge and its management. Moreover, the source of information about food allergies was also found to be significantly associated with the knowledge score and the level of awareness regarding food allergies. This study provides evidence that there is a significant influence of food allergy knowledge of parents of autistic children on its management among autistic children from Saudi Arabia, with awareness of food allergies as the mediator. Conclusions: This is the first study where the prevalence, knowledge and management, along with awareness of food allergy, has been empirically explored through the perception of parents of autistic children.

## 1. Introduction

Food allergy can be described as the “adverse health effect arising from a specific immune response that occurs reproducibly on exposure to a given food”, which varies from food intolerance as the latter is a “nonimmune reaction that includes metabolic, toxic, pharmacologic, and undefined mechanisms” [1]. The clinical response of food allergy varies from being mildly discomforting (tingling/itching) to severe life-threatening issues and even mortality of the affected individuals [2]. As shown in WHO [3], food allergies are highly common and prevalent in 1 to 3% of adults and 4 to 6% of children across the world. Over the years, the occurrence of food allergies has drastically increased, going beyond 25 to 30% [4] and, in some cases, up to 66% [5]. Even though all food items can be potentially allergenic, in general, seventy or more types of food items have been found to be causing food allergy [3]. 

The Codex Alimentarius [6] developed by WHO and the Food and Agriculture Organization (FAO) of the United Nations has validated the enlisted eight food categories, including gluten-containing cereals (such as wheat and barley), fish (shellfish), eggs, crustacean species, milk (from a cow), soybeans and dry fruits and nuts as hypersensitive food, which account for 90% of food allergens along with dairy products, eggs from a hen, legumes, fruits such as kiwis, apples, grapes in their juice form and certain vegetables such as carrots and onions also tend to contribute to allergic reactions [7]. Sesame, which was added as the ninth “food allergen recognized by the US” [8], is an important food in the Middle East. Moreover, it can be speculated that these allergens and the rate of prevalence of these allergies also seem to vary from region to region [2]. The severity and complexity of the reaction make food allergies impact the quality of life [9] and fall under the category of highly critical serious health concerns that require constant monitoring and management [5].

The health conditions become even more concerning for individuals with developmental disorders, especially autism spectrum disorders (ASD) and many others [10]. Autism has been established as a “complex neurodevelopment disorder” that impacts children even before they turn three years old [11,12]. There is a characteristic delay in language skills, speech impairment, lack of socializing skills, lag in communicating and repetitious aberrant demeanor [4]. Even though the etiology of the autistic syndrome is yet obscure [13], multiple risk factors for autism have been identified and/or suggested, which include both genetic, immunological and environmental pre-disposing factors such as chemical exposure through pollution and pesticides or pre-term birth, folic acid deficiency, maternal obesity and diabetes [11]. Some researchers have construed food allergy has been construed as the “pathogenic factor” for autism [13] and have suggested that there may be an association of autism with food allergies [14], wherein it has been construed that food allergies tend to worsen the condition by losing control of the immune system [10]. However, the pathogenicity is not universally accepted and still remains unproven. Therefore, it can be implied that this association is still a matter of intense debate as there is no consensus on this matter, and it requires further validation [12]. 

The typical symptoms also for autistic individuals remain the same as others and include gastrointestinal, sleep disorders and skin-related reactions [11,12]. Common ones are queasiness, pain in the abdomen, skin rashes, redness, asthma, loose motions, urticaria, atopic dermatitis, rhinitis, anaphylactic reaction, angioedema, etc. [4,10,13]. The contact with the food allergen causes degranulation of mast and basophil effector cells and can intensify inflammatory-induced cytokine signals arising out of white blood cells (WBC), thereby inducing severe neurological abnormalities and other health issues. Even with higher prevalence, diagnosing autistic children suffering from a food allergy is highly challenging due to their autism-related issues [4]. Unfortunately, most of the ‘not so’ severe symptoms remain undiagnosed and untreated. The immunotherapeutic management of these allergies typically involves the dietary avoidance of these food items [13,15]. To do that, the foremost step includes the assessment of knowledge and awareness of food allergies among the affected individuals. 

Studies dealing with food allergy among children from the Arabian peninsula are limited [2,5] and particularly restricted to the prevalence and awareness of food allergy [15,16,17,18]. It was observed that these studies are skewed toward narrow geographical coverage within Saudi Arabia. Furthermore, both autism and food allergy has been found to be frequently prevalent in Middle East nations [2,7,19,20]. However, there are hardly any guidelines or policies regarding the management of food allergies in Saudi Arabia [21]. Comparison of food allergy [12,13,22], as well as food intolerance [17] between autistic children and neurotypical children, have shown that food allergy was more prevalent in children with ASD. The investigation of the dietary intake showed significant differences in the frequency of food intake between the autistic and the neurotypical children, where the consumption of food items such as milk, eggs, fish, liver, meat, butter and olive oil were lower in autistic children over control [20]. However, it can be speculated that this may be due to several behavioral issues observed in autistic children. The exclusion of potential allergens is the primary treatment modality against any food allergy, irrespective of the neurological condition [1] was also found also to be beneficial in managing the behavioral issues of autistic children [13]. In contrast to the above studies, evaluating the risk factors of ASD and Attention Deficit Hyperactivity Disorder (ADHD) in a retrospective study of 134 children from Jeddah, Saudi Arabia, food as a risk factor was found to be unassociated with ASD and ADHD [11]. Similarly, in an observational study with 30 autistic boys and three autistic girls, these children did not demonstrate any level of gluten sensitivity [23]. This implies the presence of conflicting results and, thus, warrants more clarity in this regard. 

None of the studies elaborate on the perception of parents with autistic children in terms of their knowledge and awareness of the concept of food allergy, even when the autistic abdominal symptom seems to be associated with food allergies [4,12,24]. Most of the studies, even on the perception of food allergy among parents, involved only frequency and percentage analysis; there were hardly any inferential studies on this topic. The present study intends to investigate the perception of parents of autistic children living in Saudi Arabia in terms of prevalence, knowledge and awareness of food allergy. Along with this, the mediating effect of awareness on the relationship between knowledge of food allergies of parents and its management for better well-being among these children was also examined. Based on the aim of the study, the following three objectives were outlined, keeping in mind the Saudi Arabian population for our study:

1. To explore the level of prevalence of food allergy, the main types of allergens and symptoms among autistic children.
2. To estimate the level of knowledge of parents about food allergies in children with autism.
3. To understand the impact of parental knowledge of food allergy on its management to ensure healthy well-being of autistic children and mediated by awareness towards food allergy.

## 2. Materials and Methods

### 2.1. Study Design

For this study, a nationwide observational cross-sectional survey in Saudi Arabia through web-based questionnaire was formulated by the researcher. A positivistic exploratory research design employing a deductive research approach to understand the extent of food allergy management among autistic children was undertaken. The duration of the study was from April 2022 to August 2022.

### 2.2. Study Population 

The sample population intended for this study comprised 125 parents of children with autism selected using the convenient non-probability sampling technique. The inclusion criteria included consent from the parents, age group of children in the range of 2 to 18 years, and diagnosis of autism or ASD. The exclusion criteria were as follows: (a) autism related to any genetic syndrome; epilepsy; celiac problems, (b) being a single child, (c) any known food allergy, such as lactose intolerance, nut allergy, etc., or (d) children on a special diet. This research was performed in conformity with the rules of the 1975 Helsinki Declaration. Approval was attained from the ethical committee of the College of Applied Medical Sciences in Taibah University, Medina, Saudi Arabia (2022/141/207/CLN) on 10 April 2022.

### 2.3. Measures

A well-structured online questionnaire was prepared by the researchers based on the literature reviews of past studies in this area. This method was found to be appropriate because of its greater geographic distance coverage, reduced cost of traveling, and the easy maintenance of proper COVID-19 protocols during data collection [25,26]. The questionnaire consisted of five main parts: demographic information of parents and their autistic children. Within the socio-demographics of parents, their age, gender, nationality, marital status, occupation, educational level, number of family members, region of residence and any history of food allergy in the family was gathered, while for the child, information was noted about their gender, age and presence of any sibling with autism. The prevalence of food allergy among autistic children was measured by enquiring about the existence of allergic reactions in the child or their siblings diagnosed either by the parents or by the doctors, followed by the age at which the child got his first allergic reaction. Along with this, any known ways by which the child obtained the allergy, the duration of the allergy till its signs wear off, types of food allergens and the common symptoms of allergy due to food were also points of inquiry. The next set of 16 statements evaluated the level of knowledge or perception of parents on food allergies with response options of yes, no and not sure. 

A scoring system based on the right answers was employed to calculate the knowledge score. Each right answer was given a score of one. Those parents who had a score of less than or equal to 8 were considered as having poor knowledge, while individuals answering 9 to 11 statements correctly were considered to have moderate knowledge and participants who identified 12 or more correct statements fell under the category of parents with good knowledge on food allergy. Awareness of food allergy and Management of food allergy with respect to medical interventions and understanding of food labeling was measured using 5-point Likert scale, ranging from 1 = Not at all to 5 = Always. In addition to this, the most common source of information about food allergies for the parents of autistic children was also probed. The final questionnaire was also translated into Arabic for a better understanding of the participants and sent to them through their emails. Under ethical considerations, it should be observed that the consent forms were distributed by the researcher before the administration of the questionnaires. Only after receiving acceptance of consent mail was the questionnaire sent to the voluntary participants. Moreover, the participants were assured that the data would be used only for academic purposes, be kept confidential and be inaccessible to any third party other than the researcher. 

### 2.4. Data Analysis 

Statistical software (SPSS version 24) was employed to convert the collected raw data into numerical data which was subjected to statistical analyses. This statistical tool has been successfully used for obtaining meaningful inferences [27]. All the study variables were measured using frequency and percentage and chi-squared test was applied to examine the association between the study variables. The influence of knowledge of food allergy on management of food allergy was examined through linear regression analysis. Along with this, mediation analysis was conducted between knowledge of parents on food allergy of autistic children and management of food allergy, mediated through awareness. The associations were accepted to be statistically significant when the *p*-value was observed to be less than 0.05 at 95% confidence level. 

## 3. Results 

### 3.1. Characteristics of the Participating Parents

Table 1 elucidates the sociodemographic characteristics of the participating parents and their children diagnosed with ASD. Out of the total of 125 responding parents, the majority of them were 31- to 40-year-old married graduate females with Saudi nationality and working in private sector, having a family of three members with a medium-range family income living in the eastern region of Saudi Arabia. Most of them (83.2%) had a family history of food allergy. Out of the 125 autistic children, 52.8% of them were boys and the rest, 47.2% of them, were girls. More than 58% of them were within the age group of two to four years old, and the majority (82.4%) of them did not have a sibling with autism. 

### 3.2. Prevalence of Food Allergy among Autistic Children

Table 2 presents the frequency of autistic children and others with food allergies. It was observed that only 29 (23.4%) of the children with ASD suffered from a food allergy, while only 36% of the parents agreed that their other children also had some type of food allergy. Regarding the age at which food allergy was first observed in the autistic children, it was found that food allergy occurred when they were mostly one to two years old (45.1%), followed by 20.9% of them, whose first symptom was detected when they were more than six years old, while 19.8% of them claimed that their children were only three to six years old when they got their first allergic attack. Moreover, only 14.3% of them got their first allergic reaction before their first birthday. The primary mechanism of getting the food allergy was through eating (65.5%), followed by smelling (10.3%) or by touching (6.9%). About 17.2% of autistic children got their allergy from a combination of eating, smelling as well as touching. When enquired about the duration till the signs of allergy wear off, the majority (69%) of the parents construed that the allergic reactions typically last for some minutes to hours, whereas the rest (31%) of them took days to wear off the allergy. 

Table 3 gives the distribution of children with respect to the various categories of food allergens. It was observed that allergy due to consumption of food rich in proteins (18.4%) was most common among autistic children, followed by cereals (15.2%), cocoa (9.6%), fruits and nuts (8.8%) and vegetables (5.6%). Within proteins, cow milk was found to be the most frequent allergen, impacting 72.4% of the affected children, followed by seafood (69%), fish (37.9%) and chicken (20.7%). Among vegetables, beans caused allergy in 24.1% of the allergic children, followed by eggplant and tomato (17.2% each). Potato, spinach and onion were non-allergens for this set of children. Banana was the reason for allergy in 20.7% of the children, followed by strawberry (13.8%), grapes (10.3%) and apples (3.4%). Between the dry fruits, almonds, peanuts and hazelnuts equally contributed to 24.1% of the affected children and wheat and rice impacted 65.5% and 31.0% of these children, respectively. Cocoa too caused allergy in 41.4% of the 29 children who had a food allergy. No statistically significant association was observed between the demographics of the autistic children with the type of allergens (*p* > 0.5). 

The top three most common symptom of food allergy observed in autistic children was skin redness (72.4%), followed by skin rash (69.0%) and itching (62.1%). The other symptoms include shortness of breath (31.0%); swelling of body parts, especially the tongue (20.7%); abdominal pain (17.2%); nausea and vomiting (13.8% each); difficulty in swallowing and diarrhea (10.3% each) and runny nose and cough (6.9% each). 

### 3.3. Knowledge Level of Parents about Food Allergies in Children with Autism

**Figure 1:**
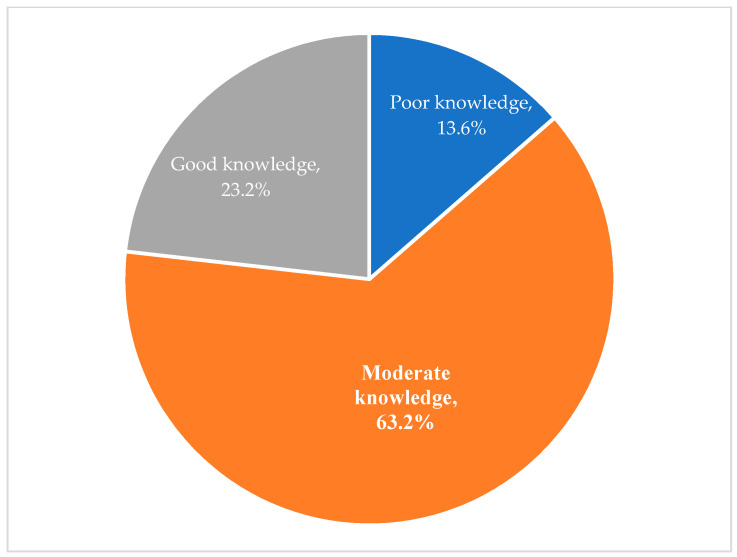
Knowledge about food allergy of parents.

The knowledge of parents of autistic children on food allergies was assessed by asking them true or false about 16 related statements (Table 4). Based on the knowledge scoring system as explained in the methodology, it was estimated that more than 62.2% of correct answers were given by the parents and the majority (63.2%) of them had a moderate level of knowledge about food allergens (score of 9 to 11 out of 16), while 23.3% had good knowledge (score of 12 to 16) and only 13.6% had poor knowledge (score of 1 to 8) on these allergies (Figure 1). 

Furthermore, the association of the knowledge score of parents with their demographics was analyzed (Table 5), and it was found to be statistically significant in terms of their age (chi-square = 34.011, *p* < 0.01), nationality (chi-square = 26.343, *p* < 0.01), educational qualification (chi-square = 17.126, *p* < 0.01), and family history of food allergy (chi-square = 51.749, *p* < 0.001). Poor knowledge was associated with young parents (18 to 30 years) and non-Saudi nationals with no family history of food allergy, which may be due to inexperience. Gender of the parent, their occupation and location of residence did not play any role in the knowledge of food allergy among these parents with autistic children. 

### 3.4. Awareness and Management of Food Allergies

With respect to food allergy awareness, the majority of parents (41.6%) felt that they were ‘somewhat’ aware of the food items that cause food allergy in their child and often felt that the food should be monitored before their child eats it (36%). The management of food allergies was evaluated through two sub-variables, such as the use of medical interventions and the understanding of food labeling. Within medical interventions, the majority of parents often kept an epinephrine pen handy (34.4%). More than 38% of them had to send their child to the emergency room (ER) or have been admitted to the hospital due to food allergies. Moreover, the parents very well knew which doctor to be consulted when their child got an allergic reaction. In terms of food labeling, more than half (52.8%) of the participants believed that food labeling is extremely helpful in avoiding known medical allergies. Almost half of them were aware of the information for labeling food allergens, while 46.4% of them were cautious about the contents in the food that was given to the child.

### 3.5. Influence of Knowledge on Management of Food Allergy Mediated by Awareness

**Figure 2:**
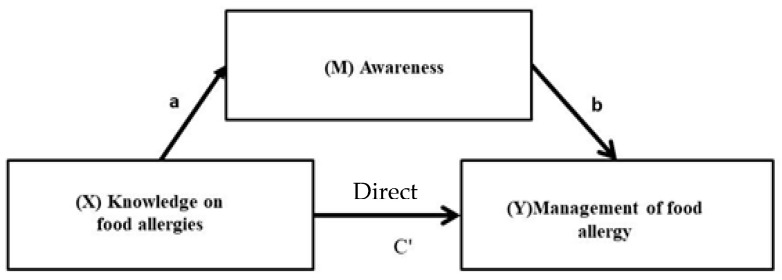
The influence of knowledge of food allergies on management of food allergy mediated by awareness.

Figure 2 summarizes the role of food allergy awareness as a mediator on the impact of knowledge on the management of food allergies. Table 6 presents the results of the mediation analysis to evaluate the total effect (C), the indirect effect (ab) and the direct effect (C’) and their associated 95% confidence intervals. According to Figure 2, the outcome variable for the analysis was the management of food allergy. The predictor variable for the analysis was knowledge of food allergies, while awareness of food allergy was evaluated as the mediator. The results show that the level of knowledge positively predicts the management of food allergy (β = 0.24 for moderate and β = 0.33 for high knowledge score of parents, *p* < 0.05) (Model 1). Analysing the indirect effects, results reveal that awareness completely mediates the relationship between knowledge and management of food allergy in parents with moderate knowledge of food allergy, but awareness of food allergy partially mediates the same relationship in parents with a high level of allergy knowledge. 

### 3.6. Source of Information about Food Allergies

In Table 7, the majority (42.4%) of the parents were dependent upon social media as the main source of information on food allergy, followed by their learnings from their education (14.4%) and the advice given by doctors (12.8%). Social media has been argued to play a critical part in enhancing the awareness of parents, thereby altering their attitude towards food allergy [17]. Significant associations were observed between the source of information with the knowledge level on food allergy (chi-square = 20.376, *p* < 0.01) and awareness of food allergy (chi-square = 19.374, *p* < 0.01). It was observed that the advice of the doctor or the health care professional e.g. dietitians was considered a better source of quality information and the best source for food allergy compared to social media.

## 4. Discussion

The findings of this study deal with knowledge and awareness of food allergy and its management among parents of autistic children in Saudi Arabia. Among the studied parental attributes, it was observed that the majority of the participating parents of the autistic children were in their middle age and non-graduates, as also observed by Alqahtani et al., [17], where the focus was on the perception of food allergy among mothers whose children suffered from food allergy. Similar to Alanazi et al., [16], both mothers and fathers equally contributed to the data collection for this study. In a separate study from Saudi Arabia, the participating parents (mothers) were housewives and much older in age than our study population [18]. 

The selected participants reported that less than one-fourth of the autistic children faced the issue of food allergy. Regarding the prevalence of food allergies in autistic children, the findings of the present study show a much higher degree of prevalence compared to the levels reported by others [12,13], where the prevalence was up to 14% and 11%, respectively, in children with autism. In another study, only 12% and 17.5% of the children from cities in Saudi Arabia were allergic to food [16,18]. Similar results were observed by Alqahtani et al., [17], where the prevalence was about 29%. Moreover, Alotaibi et al., [15] reported food allergies to be present in almost 50% of children living in Saudi Arabia. In adults, the prevalence of food allergy was restricted to 21.4% [2]. This difference may be due to the fact that food allergy varies with changes in geographical location and also has been expected to be more evident in autistic children [4]. Moreover, the observed higher frequency of food allergies was common in the first couple of years of life, as observed in our study was also reported by [17,28]. However, Alotaibi et al., [15] observed that the majority of the children were less than one year old when they had their first episode of food allergy. It should be kept in mind that increased prevalence towards food allergy among austistic children found in this study may be due to the self-reporting by the parents or the caregiver. There is a possibility of an intrisic bias, therefore, this requires further validation performed in a scientific manner. Our findings on mechanism of food allergy and duration till signs of allergy wears off were found to be similar as also observed by Alqahtani et al., [17], where eating was the primary mode of getting the allergy and the allergy stayed for minutes to hours in the children. However, Alotaibi et al., [15] found that sometimes the timing of the allergic response to develop symptoms itself can stretch beyond 24 h in rare cases. Most of them in our case had allergic symptoms within 1 to 2 h of exposure to the allergen. 

Among all food categories, the primary allergen for autistic children, as observed by their parents’ involved food rich in proteins. This can be supported by Youssef et al., [24], wherein it was reported that digesting protein is difficult for children with autism. Within proteins, milk was observed to be the primary source of allergy in the children whose parents participated in our study. This is contradictory to Alotaibi et al., [15] and Alanazi et al. [16], where fish caused food allergy in the majority of children from Saudi Arabia, followed by beans. Shellfish, followed by egg and milk, were found to be the most common allergens in children from Saudi Arabia, as observed by Alqahtani et al., [17]. However, it was also reported that egg-induced maximum food allergy in adults [2] as well as children [28] from Saudi Arabia. Moreover, WHO [3] reports on food allergies suggested that egg along with milk allergies are more common in infants compared to adults. In a separate study, peanut, coffee, crab, apple and potato were found to be highly allergenic in autistic children from India, while peanut was the primary allergen for Canadian children [14], which was completely contradictory to our observations of any form of nuts. As reported in Gomaa et al., [18], nuts are the most common type of food causing allergies in children from Saudi Arabia. 

Among the most common symptoms, redness was also observed in the majority of children as an allergic reaction by [18,28] in children from UAE and Saudi Arabia, respectively. Contradictory results were observed in terms of the most frequent symptom observed due to food allergy by [15,16], where parents of children living in the cities of Saudi Arabia reported itching to be the most frequent symptom. 

Compared to the findings of the present study, where the majority of them had moderate levels of knowledge on food allergy, the majority of mothers from a study in Saudi Arabia were found to have poor allergy knowledge [18]. The knowledge of food allergy in parents has been observed to vary drastically depending upon the food item in question, as also observed by [16]. Regarding the association studies between demographics and knowledge of food allergy, age was found to be associated with knowledge scores in a study with mothers of allergic children from Saudi Arabia [18], similar to our findings. However, in contrast to our study, the educational qualification and occupation, in this case, were unrelated to knowledge levels.

Contrary to our findings on the level of food allergy awareness, [15,17] reported conflicting observations in this regard among the Saudi population. Gomaa et al., [18] suggested that the knowledge and awareness were both poor in Saudi Arabian mothers. Apart from this, even the awareness of pediatricians in Kuwait on food allergy was also observed to be limited [29]. As reported in Alotaibi et al., [15], the majority of the parents did not seek much medical advice regarding food allergy even after the symptoms were observed. The use of epinephrine is not quite common among the Saudi Arabia population as the awareness about it seems to be low [19,30]. People, even school authorities or teachers, hardly know about this medication. Even though the majority of the parents in our study were aware of the food allergen in their child, the management levels towards controlling or avoiding allergies were found to be poor. This was also implied by [31], where food allergen knowledge was negatively but insignificantly related to food allergy management practices.

A significant influence of knowledge of food allergy of parents on the management of food allergy was observed, which was mediated by awareness of food allergy. The mediation analysis showed that parents with good knowledge of food allergy significantly influence food allergy management in autistic children. However, parents with a moderate level of knowledge have hardly any impact on allergy management. Therefore, awareness in this aspect is essential, especially for the population who have a moderate level of knowledge of food allergies. As expected, the management can be better controlled by enhancing knowledge and awareness of it. Therefore, raising awareness of food allergy is required for Saudi society, especially with regard to autistic children, as also realized by [19]. In fact, not only autistic children; WHO [3] has pointed out that awareness in this aspect is the “1st step in protecting individuals with food allergies” among all. Moreover, the criticality of this issue and the need for enhancing awareness of food allergies among the general population has also been reported from studies of other countries [7,9,31,32], as the data on this topic is “scarce” [2]. Unfortunately, none of the studies have done similar mediation analyses in this aspect; therefore, our findings could not be compared much with others. 

Supporting our results from this study, social media has been argued to play a critical part in enhancing the awareness of parents, thereby altering their attitude towards food allergy [17]. Regarding the source of information, our results matched with the perception of parents of children from Saudi Arabia suffering from food allergies, as also described by [16], where the internet was responsible for the spread of knowledge on possible allergens, followed by books or magazines. However, this study did not evaluate the knowledge level involving food allergy. Our results, however, are in contrast with Soon [31], where the participants from the UK did not apply social media to enhance their knowledge of food allergens. 

Even though this has been one of the pioneering contributions to the outlook involving food allergies in autistic children, this study was restricted in terms of sample size, the scope of bias and misreporting as this study was based on self-reporting rather than through clinical guidelines. Moreover, no immunological tests were conducted to actually assess the immune response of the affected children. In addition, no neurotypical children were considered for the study. Moreover, the degree of autism was also not studied during the course of the study. Therefore, increasing the sample size, obtaining validation from doctors about the genuine nature of food allergy, and comparing it with non-autistic children from the same area can lead to an in-depth analysis of the issue, which will be helpful in obtaining the whole picture as a part of future research. Furthermore, research should also focus on finding easier methods for identifying food allergies and understanding the various aspects of food allergy management, which will be beneficial in managing ASD as well. 

## 5. Conclusions and Future Scope

The present study explored the prevalence, awareness and management of food allergies through the perceptions of parents with autistic children and showed that food allergy can be a major problem among autistic children, even though this is yet to be supported by scientific evidence. A high prevalence of food allergies was observed in these children, thereby implying that it is crucial to spread awareness of food allergens among the population in order to have better management of reactions to food allergies. Moreover, this study also elaborates on the types of food allergens and the symptoms observed during allergies in autistic children. Since the overall knowledge among the majority of parents was found to be moderate, an increase in knowledge is urgently warranted, especially among parents of children with developmental disorders. Moreover, awareness was found to completely mediate the relationship of knowledge on food allergy management in terms of parents with a moderate level of knowledge, while for parents with a high level of food allergy knowledge, awareness only partially mediated the relationship. From our study, it can be suggested that awareness of food allergy should take precedence by the healthcare ministry. Moreover, the healthcare agencies, as well as the healthcare ministries, should start intensive programs that ensure an increase in correct and accurate knowledge about food allergies. Our results emphasize that food allergy management is not being considered seriously among this kind of population, which is a matter of concern not only for the patients but also for their caregivers, the government, doctors, hospitals and society as a whole. This task is essential for ensuring the healthy well-being of autistic children since these children are more affiliated to be impacted by food allergies compared to children who do not have ASD. However, this study in no way implies that food allergy is the causal agent of ASD among children and, therefore, needs to be confirmed through medical analysis. To the best of our knowledge, this is the first study exploring the awareness and management aspects regarding food allergy in children with autism as well the role of awareness as a mediator, where knowledge influences food management. Another key strength of this study includes the inclusion of participants from all regions of Saudi Arabia.

## Figures and Tables

**Table 1:** Characteristics of the participating parents and children.

Demographics	Categories	Frequency	Percent
**Sociodemographic characteristics of the parents**			
**Age (years)**	18–30	18	14.4
	31–40	71	56.8
	>50	36	28.8
**Gender of parent**	Male	54	43.2
	Female	71	56.8
**Nationality**	Saudi	101	80.8
	Non-Saudi	24	19.2
**Marital status**	Married	103	82.4
	Separated/Divorced/Widowed	22	17.6
**Family members**	1	16	12.8
	2	26	20.8
	3	77	61.6
	>4	6	4.8
**Family income**	Low	16	12.8
	Medium	57	45.6
	High	52	41.6
**Occupation**	Homemaker	10	8.0
	Private Sector	61	48.8
	Governmental Sector	54	−43.2
**Educational qualification**	Low educate	6	4.8
	High school	41	32.8
	Diploma	26	20.8
	Graduate	52	41.6
**Region of residence**	Northern	20	16.0
	Eastern	48	38.4
	Southern	12	9.6
	Western	22	17.6
	Central regions	18	14.4
	Any other	5	4.0
**Family history of food allergy present**	No	18	14.4
	Yes	104	83.2
	Unsure	3	2.4
**Demographic characteristics of the autistic child**			
**Gender of the autistic child**	Boy	66	52.8
	Girl	59	47.2
**Age of the child with autism(years):**	2–4	73	58.4
	5–7	27	21.6
	8–12	16	12.8
	13–17	9	7.2
**Any sibling with autism**	No	103	82.4
	one or more than one	22	17.6

**Table 2:** Prevalence of food allergy in children.

Prevalence of Food Allergy	No	Yes	Unsure
**My child with ASD is allergic to some food items**	88 (70.8)	29 (23.4)	8 (6.4)
**One of my children has some type of food allergy**	66 (52.8)	45 (36.0)	14 (11.2)

**Table 3:** Distribution of autistic children in terms of types of food allergens and their demographics.

Demographics of Children		Type of Allergen, Frequency (Percent)				
		Proteins	Vegetables	Fruits and Nuts	Cereals	Cocoa
**Gender of the autistic child**	Boy	10 (15.2)	2 (3)	4 (6.1)	8 (12.1)	4 (6.1)
	Girl	13 (22)	5 (8.5)	7 (11.9)	11 (18.6)	8 (13.6)
	Chi sq., *p* value	0.983, 0.322	1.747, 0.186	1.307, 0.253	1.028, 0.311	2.018, 0.155
**Age of the child with autism (years):**	2–4	15 (20.5)	3 (4.1)	6 (8.2)	11 (15.1)	8 (11)
	5–7	5 (18.5)	2 (7.4)	3 (11.1)	4 (14.8)	2 (7.4)
	8–12	1 (6.3)	0 (0)	0 (0)	1 (6.3)	0 (0)
	13–17	2 (22.2)	2 (22.2)	2 (22.2)	3 (33.3)	2 (22.2)
	Chi sq., *p* value	1.885, 0.597	6.127, 0.106	3.775, 0.287	3.294, 0.348	3.656, 0.301

**Table 4:** Knowledge of parents on food allergy.

	Knowledge of Food Allergy	Correct Answer	Frequency (Percent)		
			No	Yes	Unsure
**1.**	Food allergy implies that food is harmful	Yes	78 (62.4)	43 (34.4)	4 (3.2)
**2.**	Lactose and gluten intolerance is not related to food allergy	Yes	43 (34.4)	76 (60.8)	6 (4.8)
**3.**	I know some food items can cause allergy	Yes	22 (17.6)	95 (76)	8 (6.4)
**4.**	Food allergies are mostly spread to the child through breastfeeding	No	96 (76.8)	22 (17.6)	7 (5.6)
**5.**	An allergic reaction can get triggered just by touching the food	Yes	107 (85.6)	18 (14.4)	0 (0.0)
**6.**	Food allergies are more common in children compared to adults	Yes	26 (20.8)	88 (70.4)	11 (8.8)
**7.**	Medicine needs to be taken regularly in order to prevent food allergy	Yes	87 (69.6)	32 (25.6)	6 (4.8)
**8.**	Food allergy can be dangerous for the child; therefore, it needs serious consideration	Yes	18 (14.4)	105 (84)	2 (1.6)
**9.**	Avoiding the allergenic food can prevent my child from getting food allergy	Yes	39 (31.2)	80 (64)	6 (4.8)
**10.**	Food allergy can be solved only by drug therapy	No	93 (74.4)	25 (20)	7 (5.6)
**11.**	The child needs to be taken to the hospital if they develop the food allergy	Yes	47 (37.6)	75 (60)	3 (2.4)
**12.**	Food allergy is mostly hereditary	Yes	42 (33.6)	74 (59.2)	9 (7.2)
**13.**	Food allergy is an infectious disease	No	81 (64.8)	39 (31.2)	5 (4)
**14.**	Allergy towards food is a chronic issue	Yes	25 (20)	91 (72.8)	9 (7.2)
**15.**	Food allergy can be determined by blood tests	Yes	25 (20)	94 (75.2)	6 (4.8)
**16.**	A medical alert bracelet can make other people aware of food allergies	Yes	0 (0.0)	103 (82.4)	22 (17.6)

**Table 5:** Association of knowledge score about food allergy and demographics of the parents.

Demographics	Categories	N	Knowledge of Food Allergy			*p* Value
			Poor	Moderate	Good	
**Age (years)**	18–30	18	10 (55.6%)	6 (33.3%)	2 (11.1%)	0.000 *
	31–40	71	4 (5.6%)	52 (73.2%)	15 (21.1%)	
	>50	36	3 (8.3%)	21 (58.3%)	12 (33.3%)	
**Gender of parent**	Male	54	9 (16.7%)	31 (57.4%)	14 (25.9%)	0.480
	Female	71	8 (11.3%)	48 (67.6%)	15 (21.1%)	
**Nationality**	Saudi	101	6 (5.9%)	70 (69.3%)	25 (24.8%)	0.000 *
	Non-Saudi	24	11 (45.8%)	9 (37.5%)	4 (16.7%)	
**Educational qualification**	Low education	6	4 (66.7%)	2 (33.3%)	0 (0.0%)	0.009 *
	High school	41	4 (9.8%)	25 (61%)	12 (29.3%)	
	Diploma	26	2 (7.7%)	17 (65.4%)	7 (26.9%)	
	Graduate	52	7 (13.5%)	35 (67.3%)	10 (19.2%)	
**Region of residence**	Northern	20	3 (15.0%)	14 (70.0%)	3 (15.0%)	0.260
	Eastern	48	9 (18.8%)	29 (60.4%)	10 (20.8%)	
	Southern	12	1 (8.3%)	8 (66.7%)	3 (25%)	
	Western	22	1 (4.5%)	16 (72.7%)	5 (22.7%)	
	Central regions	18	1 (5.6%)	9 (50%)	8 (44.4%)	
**Family history of food allergy present**	No	18	11 (61.1%)	7 (38.9%)	0 (0.0%)	0.000 *
	Yes	104	4 (3.8%)	71 (68.3%)	29 (27.9%)	
	Unsure	3	0 (0.0%)	2 (66.7%)	1 (33.3%)	
**Occupation**	Not working	10	2 (20%)	4 (40%)	4 (40%)	0.252
	Private Sector	61	11 (18%)	38 (62.3%)	12 (19.7%)	
	Governmental Sector	54	4 (7.4%)	37 (68.5%)	13 (24.1%)	

* *p* < 0.01.

**Table 6:** Effect of mediation by awareness on relationship between knowledge and management of food allergy.

Knowledge	Model 1 (Estimating C)		Model 2 (Estimating a)		Model 3 (Estimating b and C’)	
	Management of Food Allergy		Awareness		Management of Food Allergy	
	95% CI	β	95% CI	Β	95% CI	Β
**Moderate**	−4.508–−0.044	0.247 *	−0.886–−0.811	0.483 *	−0.260–0.067	0.996
**High**	−5.387–−0.136	0.337 *	−0.844–−0.466	0.445 *	−0.359–−0.036	0.198 *
**Low**	1	Ref	1	Ref	1	Ref

* *p* < 0.05.

**Table 7:** Association of source of information about food allergy with knowledge score and awareness of food allergy.

Common Sources of Information	Frequency (Percent)	Knowledge of Food Allergy			Awareness of Food Allergy		
		Poor	Moderate	Good	No	Yes	Unsure
**Academic curriculum**	18 (14.4)	1 (5.6%)	14 (77.8%)	3 (16.7%)	3 (16.7%)	15 (83.3%)	0 (0.0%)
**Books or magazines**	17 (13.6)	3 (17.6%)	8 (47.1%)	6 (35.3%)	4 (23.5%)	11 (64.7%)	2 (11.8%)
**Social media**	53 (42.4)	6 (11.3%)	32 (60.4%)	15 (28.3%)	20 (37.7%)	31 (58.5%)	2 (3.8%)
**Educational lectures**	9 (7.2)	3 (33.3%)	2 (22.2%)	4 (44.4%)	3 (33.3%)	6 (66.7%)	0 (0.0%)
**Previous experience**	12 (9.6)	3 (25%)	8 (66.7%)	1 (8.3%)	2 (16.7%)	8 (66.7%)	2 (16.7%)
**Doctor or health care professional**	16 (12.8)	1 (6.3%)	15 (93.8%)	0 (0%)	0 (0.0%)	16 (100%)	0 (0.0%)
	*p* = 0.026 *				*p* = 0.036 *		

* *p* < 0.01.

## Data Availability

Not applicable.
